# Incremental peritoneal dialysis: less is more—for the patient and the planet

**DOI:** 10.3389/fmed.2025.1676396

**Published:** 2025-10-08

**Authors:** Luca Nardelli, Antonio Scalamogna, Carlo Alfieri, Federico Alberici, Giuseppe Castellano

**Affiliations:** ^1^Fondazione IRCCS Ca' Granda Ospedale Maggiore Policlinico, Milan, Italy; ^2^Department of Clinical Sciences and Community Health, Universita degli Studi di Milano, Milan, Italy; ^3^Azienda Socio Sanitaria Territoriale degli Spedali Civili di Brescia, Brescia, Italy; ^4^Department of Medical and Surgical Specialities, Radiological Sciences and Public Health, Universita degli Studi di Brescia, Brescia, Italy

**Keywords:** peritoneal dialysis, incremental peritoneal dialysis, green nephrology, environment, carbon footprint, dialysis waste, plastic waste, water usage

## Abstract

Climate change poses growing threat to global health, and paradoxically, the health-care sector—including nephrology—is a significant contributor to greenhouse gas (GHG) emissions. Dialysis, in particular, is resource-intensive. Yet, dialysis remains life-saving for over 4 million people globally, a number projected to rise sharply. While peritoneal dialysis (PD) offers a home-based alternative to hemodialysis with potentially lower environmental costs, it still generates considerable carbon emissions and waste—especially from the production, packaging, and transport of dialysate solutions. A typical continuous ambulatory PD patient generates over 600 kg of waste per year, much of it non-biodegradable polyvinyl chloride. PD’s carbon footprint ranges from 1.2 to 4.5 tons of CO₂-equivalent annually, primarily from packaging and transport. Incremental peritoneal dialysis (iPD)—an approach that starts therapy at a reduced dose based on residual kidney function (RKF)—offers a more sustainable model. Incremental PD reduces water usage, plastic waste, and carbon emissions by as much as 30–45% compared to full-dose PD. Clinically, iPD is associated with better quality of life, fewer infections, less glucose exposure, and potential preservation of RKF. Economically, it offers substantial cost savings, with estimates up to €8,700 saved annually per patient. Despite its benefits, barriers to iPD adoption include provider unfamiliarity, patient reluctance to intensify treatment, reimbursement limitations, and the need for close RKF monitoring and clinical assessment. Addressing these challenges through policy reform, education, and digital tools could enable broader implementation of iPD, aligning kidney care with environmental stewardship.

## The environmental cost of dialysis

Climate change presents an escalating global health threat, and ironically, the health-care sector itself is a major contributor to the environmental crisis. In 2013, over 10% of the United States’ total greenhouse gas (GHG) emissions were attributed to health-care activities, with similarly significant contributions in Australia (7% in 2014–2015) and the United Kingdom (4% in 2015, despite targeted carbon-reduction initiatives) (1–3). If considered as a standalone nation, the global health-care sector would rank as the fifth largest emitter of GHGs being now responsible for approximately 4–6% of global emissions (4).

Among medical specialties, nephrology—particularly the provision of dialysis—has a disproportionately high environmental impact (5–7). Dialysis treatments are resource-intensive, involving large volumes of water, significant energy consumption, and the generation of considerable amounts of single-use plastic waste (3, 8–10). Hemodialysis (HD), the most widely used renal replacement therapy, is estimated to produce between 24.5 and 65.1 kg carbon dioxide equivalent (CO₂-eq) emissions per treatment. On an annual basis, this equates to 3.8 to over 10 metric tons of CO₂-eq per patient (11, 12).

**Figure 1:**
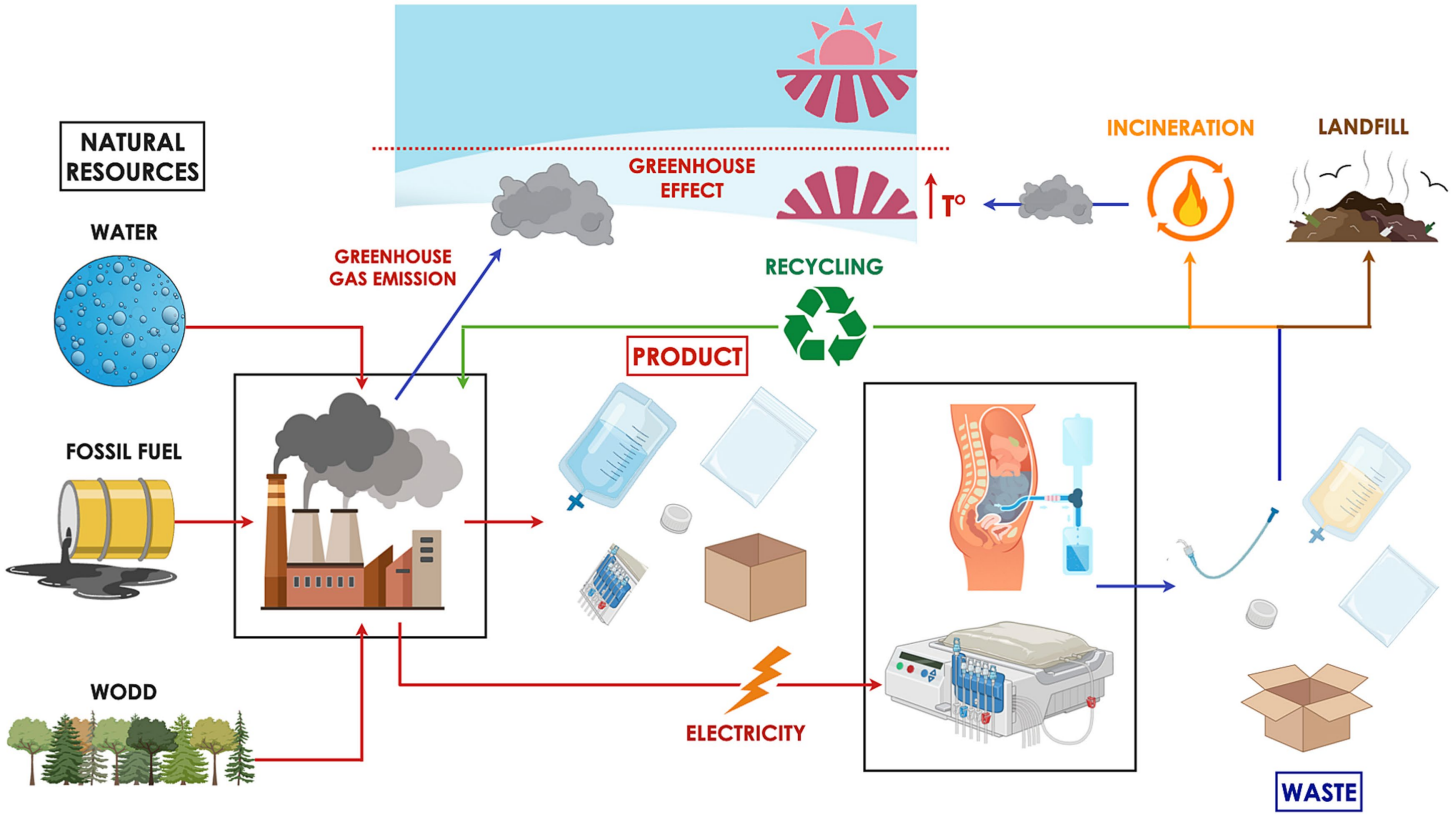
Peritoneal dialysis (PD) has a considerable environmental footprint. The treatment relies on natural resources such as fossil fuels, water, and wood for the manufacture of necessary supplies and the energy required to operate equipment. The industrial production process emits greenhouse gases that trap solar heat, contributing to the warming of the Earth’s surface and lower atmosphere. Waste generated from PD is typically categorized as hazardous, general, or recyclable. Hazardous waste, due to its potential for infection transmission, is incinerated or chemically sterilized prior to landfill disposal—procedures that are both environmentally harmful and financially burdensome. General waste, often sent directly to landfill, poses long-term environmental risks by leaching toxins into soil and groundwater. Meanwhile, the feasibility of recycling medical waste varies significantly across countries, depending on infrastructure, regulatory policies, and associated costs.

Peritoneal dialysis (PD), while often performed at home and potentially less resource-intensive on a per-treatment basis, also contributes substantially to environmental deterioration —particularly due to the production, packaging, and transport of large volumes of pre-packaged sterile dialysate (Figure 1) (13–15).

However, dialysis is a life-saving therapy for over 4 million people worldwide, with numbers expected to exceed 6 million by 2030 due to aging populations, diabetes, and hypertension (16). While critical for survival, when these numbers are considered in the light of the current scarcity of resources and climate changes, the enormity of the economic and environmental impact of kidney replacement therapy becomes evident (3).

The concept of green nephrology has emerged in response to this challenge, urging clinicians and policymakers to mitigate the environmental impact of dialysis without compromising quality of care (Table 1).

**Table 1:** Barriers to the development of incremental peritoneal dialysis programs, along with potential solutions and necessary clinical practice implementations.

Barriers	Solutions	Clinical practice implementations
Patient reluctance to escalate the dialysis dose	Timely anticipation of treatment strategy	Implementation of numerous pre-treatment visits
Risk of fluid overload and inadequate clearance	Close clinical surveillance	Closer monitoring of weight, Kt/V, diuresis volume and residual kidney function
	Implementation of tele-health medicine	Exploitation of digital PD platforms to enhance distance monitoring
Limited provider familiarity with the dialytic approach	Proposal of educational opportunities	Allow health-care staff to spend periods in highly experienced center
	Enhancement of clinical tools	Elaboration of guiding PD protocols
Unfavourable reimbursement policy	Payment modalities riform	Concrete incentives for units with high percentage of patients on iPD

PD, peritoneal dialysis; iPD, incremental peritoneal dialysis.

## The environmental impact of peritoneal dialysis

### Water usage

Peritoneal dialysis generally requires substantially less dialysate volume than HD, with daily usage ranging from as little as 2 liters to as much as 20 liters per patient, depending on the individual prescription and clinical need (17). However, analogous to HD, the production of each liter of ultrapure dialysate necessitates the use of multiple liters of source water, as purification via reverse osmosis and deionization typically consumes 2–3 liters of raw water for every liter of ultrapure water generated (18).

In addition, PD dialysate is supplied in pre-packaged sterile containers, usually composed of plastic. The water footprint associated with plastic production adds further to the environmental impact (Figure 1). Although the exact water requirement varies based on the type and manufacturing method of the plastic, it is estimated that the production of 1 kilogram of plastic consumes approximately 180 liters of water (19). An empty 2-liter PD dialysate bag weighs approximately 155 grams, implying that the manufacturing process for each bag may require an estimated 28 liters of water, based on standard water usage for plastic production. Consequently, the cumulative water burden of PD includes not only the dialysate volume itself but also the indirect water consumption embedded in packaging materials (Table 2).

**Table 2:** Characteristics of incremental, low-clearance, palliative, and decremental peritoneal dialysis strategies.

Strategy	Purpose	Prescription	Target population
Incremental Peritoneal Dialysis	To provide adequate clearance while leveraging residual kidney function; gradually intensify as RKF declines.	Reduced initial dose (fewer exchanges, lower volumes, or fewer treatment days per week), with stepwise escalation as RKF diminishes.	Incident peritoneal dialysis patients with preserved residual kidney function, suitable for a gradual start of dialysis.
Low Clearance Peritoneal Dialysis	To deliver partial solute clearance at minimal cost; not designed for dose escalation.	Fixed reduced dose, often with fewer exchanges without progressive intensification.	Patients in low income countries where resource limitations constrain therapy.
Palliative Peritoneal Dialysis	To prioritize comfort, symptom control, and quality of life over clearance adequacy.	Less aggressive regimens tailored to minimize treatment burden (e.g., fewer daily exchanges or smaller volumes).	Frail, elderly, or highly comorbid patients.
Decremental Peritoneal Dialysis	To gradually reduce treatment intensity as part of end-of-life care, aligning with patient comfort.	Stepwise reduction in exchanges and volumes, aiming to minimize invasiveness rather than maintain clearance.	Patients in terminal phases of illness, or approaching end of life. Transitioning to conservative or comfort-focused care.

### Waste generation

Health-care systems generate substantial quantities of waste, which can be broadly classified into three main disposal streams: hazardous (infectious) waste, general waste, and recyclable waste (20). Hazardous waste, due to its potential to transmit infection, must be incinerated or chemically sterilized before landfill disposal—processes that are both environmentally damaging and financially costly. General waste, which typically goes directly to landfill, can pose long-term environmental risks. For instance, toxins such as phthalates, commonly found in medical-grade plastics, may leach into soil and groundwater, creating persistent ecological and human health hazards (21). Additionally, organic components of landfill waste emit methane—a greenhouse gas with approximately 20 times the global warming potential of CO₂ (22). Although recycling offers an effective way to reduce the consumption of raw materials and energy compared to producing products from virgin resources, the feasibility of recycling medical waste varies widely between countries due to differences in infrastructure, policy, and cost.

Considering the two main PD providers: Vantive (formerly Baxter Kidney Care) primarily uses polyvinyl chloride (PVC) softened with plasticizers, such as Di(2-ethylhexyl) phthalate (DEHP) for dialysate and drain bags, as well as for most tubing and connectors. Caps and protective shells are typically made of polypropylene.

Fresenius, in contrast, markets a proprietary PVC-free multilayer material called Biofine®, based on polyolefins (polypropylene [PP] and polyethylene [PE] blends), for both dialysate and drain bags. Tubing is also Biofine-based, while caps and shells are commonly polypropylene. This shift is intended to reduce the environmental impact of incineration and avoid exposure to DEHP.

In addition to the bag and tubing materials, PD systems also include plastic overwraps used to protect dialysate and drain bags during storage and transport. These overwraps are typically made of PP, PE, or multilayer polyolefin laminates that can withstand sterilization while maintaining sterility. Although non-hazardous and theoretically recyclable, they usually enter the general waste stream, contributing significantly to single-use plastic waste. In contrast, the caps and connector shells are most often manufactured from polypropylene or similar medical-grade plastics but are individually packaged in paper overwraps, which reduces (though does not eliminate) the associated plastic burden. Taken together, these components—bags, tubing, overwraps, caps, and shells—account for the bulk of the material footprint of PD treatment, with limited recycling pathways available in most healthcare systems.

Data specifically quantifying waste generated from PD are limited but growing (Figure 1). A UK-based study reported that patients on continuous ambulatory peritoneal dialysis (CAPD) performing four exchanges per day generated approximately 1.69 kg of solid waste daily (20). This translates into an annual total of 617 kg of waste per patient, with more than half—about 343 kg—PVC plastic. Further insights come from a Canadian single-center study, which quantified the amount of recyclable, non-biohazardous plastic waste produced by home dialysis therapies (15). In CAPD, patients performing four daily exchanges generated an average of 58.76 grams of polypropylene and 222.88 grams of PVC plastic waste per day. This equates to an annual waste footprint of approximately 21.4 kg of recyclable PP and 81.4 kg of recyclable PVC plastic per patient.

These discrepancies may reflect several factors, including variation in methodologies for measuring and classifying waste streams, differences in local disposal practices, and, importantly, the use of different PD systems and consumables. For instance, some providers continue to rely on PVC bags and tubing softened with DEHP, while others have transitioned to PVC-free. Such brand-related material differences likely contribute to the wide range of PVC waste estimates reported in the literature.

In automated peritoneal dialysis (APD), the waste burden was even higher. Patients undergoing four nightly exchanges with a daytime fill produced an average of 81.53 grams of recyclable PP and 297.94 grams of PVC per day, resulting in annual totals of 29.76 kg and 108.75 kg, respectively. These values varied slightly depending on the specific cycler machine used. Collectively, these findings highlight the substantial and underappreciated environmental burden of PD-related plastic waste. In fact, plastics used in medical therapies are derived from fossil hydrocarbons, and their life cycle—regardless of whether they are disposed of via landfill, incineration, or recycling—ultimately results in CO₂ emissions. These emissions contribute to global warming at multiple stages, from production to decomposition.

### Carbon footprint

Carbon footprint studies assess the total amount of CO₂ emissions—both direct and indirect—associated with a specific activity or accumulated across the life cycle of a product. When evaluating the environmental impact of PD, relevant factors include energy consumption, water use, dialysate fluid manufacturing, transportation of supplies, and waste disposal (Figure 1). These activity data are typically converted into a standardized metric of tones of CO₂ equivalents (tCO₂-eq) using established emission factors. To provide context, a passenger car traveling 15,000 km per year with an average fuel consumption of 6 liters of gasoline per 100 km produces approximately 2.1 metric tons of CO₂ annually.

A single-center study in China evaluated patients undergoing CAPD with a daily dialysate dose of 8 liters (14). The analysis revealed that approximately 80% of PD’s carbon footprint was attributable to packaging materials, including plastic dialysate bags, outer packaging, and cardboard boxes. Electricity consumption and waste disposal accounted only for 5, 6 and 8% of the emissions, respectively. The total annual carbon footprint of PD was estimated at 1.4 tCO₂-eq per patient. However, this study did not account for emissions associated with pharmaceutical use or the transportation of PD fluids from the manufacturer to the point of care, and it excluded patients on APD.

In contrast, a more comprehensive Australian analysis incorporated these additional variables and included both CAPD and APD modalities (13). The annual per-patient carbon emissions related to consumables were estimated at 1.245 tCO₂-eq for CAPD and 1.992 tCO₂-eq for APD. When transportation factors were included, the total emissions for APD ranged from 2.35 to 4.503 tCO₂-eq, depending on the patient’s geographic location, whereas CAPD ranged from 1.455 to 2.716 tCO₂-eq. The greater environmental burden associated with APD was attributed to both the increased production and disposal of its consumables, as well as the higher transport-related emissions due to the greater weight and volume of fluids and supplies.

## The role of incremental dialysis

Incremental dialysis is a personalized approach to initiating dialysis in patients end-stage kidney disease patients with RKF (23). In those patients full-dose dialysis is not strictly necessary to achieve clearance targets (24). Thus, unlike the standard thrice-weekly HD regimen, four CAPD daily exchanges or every night APD treatment, incremental dialysis starts with a less frequent or lower dose of dialysis. Incremental dialysis offers a compelling pathway to sustainability. This concept applies to both HD and PD, but incremental peritoneal dialysis (iPD) is especially promising due to its adaptability, feasibility in home settings, and reduced reliance on high-tech infrastructure (25).

## The strategy of incremental peritoneal dialysis

Since the best waste is the waste that is never produced, a planetary health approach to kidney care should prioritize prevention and the maximization of transplantation or non-dialysis conservative management. However, when dialysis becomes necessary, optimal stewardship of resources should include consideration of iPD. Importantly, iPD does not imply the premature initiation of dialysis; rather, it reflects the principle that, once the clinical need for dialysis arises, treatment can begin with a reduced prescription, as a full-dose regimen is often unnecessary at the outset.

Pragmatically, the concept of iPD involves initiating peritoneal dialysis at a lower-than-standard dose, leveraging RKF in conjunction with peritoneal clearance to achieve adequate solute removal (23, 24). As RKF gradually declines or clinical indications evolve, the dialysis prescription is correspondingly intensified by adjusting the number and/or volume of daily exchanges, as well as dwell times.

While the operational definitions of iPD vary across the literature, the strategy is best understood as a dynamic and individualized treatment approach rather than a fixed prescription (23). The essential principle is that the dialysis dose alone is insufficient to meet clearance targets; rather, total adequacy depends critically on the combined contribution of both peritoneal and residual renal function (24).

Practical examples of iPD regimens include (26–31): (1) continuous ambulatory peritoneal dialysis (CAPD) with fewer than four daily exchanges, dialysate volumes of less than 2 L, or treatment delivered fewer than seven days per week; and (2) automated peritoneal dialysis (APD) performed less than 7 days weekly, with total daily volumes under 10 L and/or without a long dwell (2). Importantly, these prescriptions must be goal-directed and tailored to individual patient needs. Although traditional adequacy metrics such as a weekly Kt/V of 1.7 or creatinine clearance of 50 L/week are often referenced, the true objective of iPD is to preserve clinical well-being, focusing on control of uremic symptoms, fluid status, nutritional health, and quality of life (32, 33).

**Figure 2:**
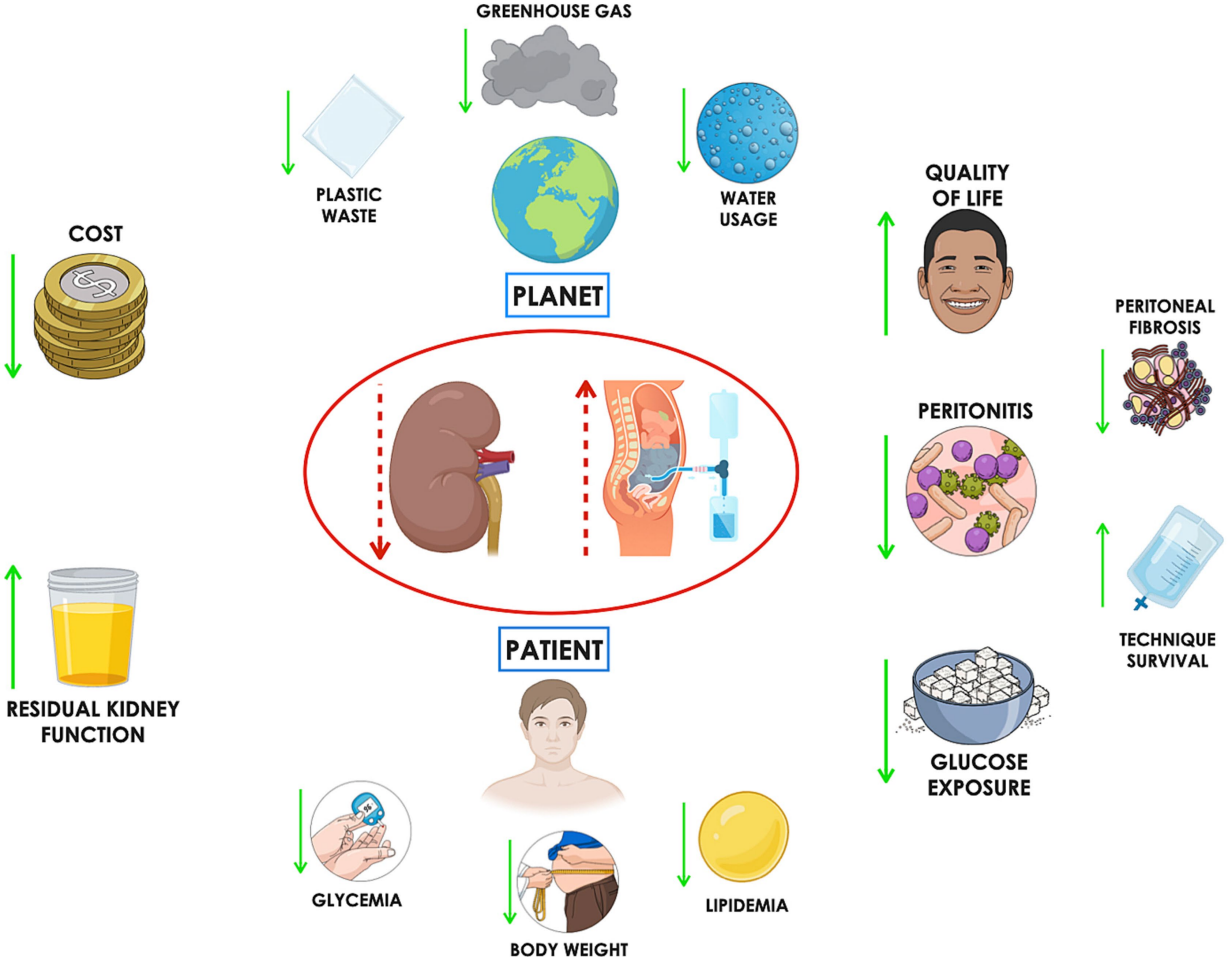
Incremental peritoneal dialysis (iPD) offers several patient-centered benefits, including increased free time, which can lead to improved quality of life and better treatment adherence. Reduced frequency of access to the peritoneal cavity may lower the risk of peritonitis. Additionally, decreased glucose exposure can help mitigate systemic risks such as weight gain, hyperglycemia, and dyslipidemia, while locally slowing the progression of peritoneal fibrosis—potentially extending technique survival. From an environmental perspective, iPD can reduce water consumption, plastic waste, and greenhouse gas emissions. Finally, the associated cost savings represent another important advantage.

By initiating therapy with a reduced dose of dialysis tailored to a patient’s RKF, iPD may delay the need for full-dose treatment, thereby mitigating the cumulative resource and emissions burden of dialysis (25) (Figure 2).

The adoption of iPD shows marked regional variability, with the most robust data available from Italy, where national registry and congress report document a steady increase in iPD use among incident patients (34). Unpublished data presented at a recent Italian national PD congress—encompassing all public PD centers in Italy—revealed a steady rise in iPD use among incident patients, increasing from 11.9% in 2005 to 40.2% in 2024 (35).

A study analyzing data from Australian and New Zealand dialysis registries found that the use of IPD among incident PD patients increased from 2.7% in 2007 to 11.1% in 2017, highlighting growing clinical interest in iPD (36); while in Japan and South Korea, several cohort studies confirm its routine application, particularly in patients initiating therapy with ≤4 L/day (37–39). Evidence from China and other East Asian countries largely stems from single-center feasibility and outcomes studies (40), whereas in Europe and North America, published data are more fragmented and limited to individual centers or programmatic experiences (41–43). Importantly, the lack of standardized definitions across studies—ranging from reduced daily volumes to fewer weekly APD sessions—complicates comparisons across regions. Taken together, these reports indicate that while iPD is practiced internationally and increasingly recognized as both clinically and environmentally advantageous, systematic national prevalence data outside Italy and Australia/New Zealand remain scarce, underscoring the need for harmonized reporting within dialysis registries worldwide.

## Low clearance, palliative and decremental peritoneal dialysis

While several PD strategies also begin with a reduced dialysis dose, they pursue fundamentally different objectives (Table 1). Incremental PD (iPD) is a proactive, goal-directed approach designed to progressively intensify dialysis as residual kidney function declines (23). In contrast, in some developing countries, low-clearance PD is prescribed mainly for economic or logistical reasons, providing partial solute clearance at minimal cost without stepwise intensification (44). Similarly, in frail older patients with multiple comorbidities, palliative PD may be employed, where the aim is not full clearance adequacy but rather the prioritization of symptom relief, comfort, and quality of life through a less aggressive regimen (45).

As populations age worldwide, the proportion of elderly and frail patients requiring kidney replacement therapy is increasing. In these individuals, treatment goals often extend beyond achieving biochemical targets to emphasize functional preservation, symptom management, independence, and overall quality of life. Palliative PD aligns well with these priorities for several reasons. First, it allows a tailored initiation of dialysis that leverages residual kidney function to meet clinical needs without imposing the full procedural burden of standard regimens. For frail or very elderly patients, this frequently translates into fewer daily exchanges, reduced catheter manipulations, and lower treatment-related fatigue, thereby supporting functional capacity and adherence. Second, reduced glucose exposure and lower dialysis intensity may mitigate metabolic complications and slow peritoneal membrane injury. Third, the possibility of home-based delivery decreases caregiver strain, reduces hospital-associated risks (including infection and deconditioning), and aligns with the preference for conservative, home-centered care often expressed by older patients. Another related concept is decremental PD, a strategy in which dialysis is deliberately reduced in frequency or intensity over time, typically in the setting of diminishing clinical benefit or at the end of life, with the aim of minimizing treatment burden while maintaining partial symptom control.

Ultimately, by lowering treatment burden, conserving resources, and reducing environmental impact, incremental, low-clearance, palliative and decremental PD align clinical care with the principles of environmental stewardship (Table 1).

## Environmental advantages of incremental peritoneal dialysis

### Reduced water usage

Incremental peritoneal dialysis significantly reduces dialysate volume by initiating therapy with fewer daily exchanges. For example, a patient prescribed two exchanges per day or five automated treatments per week typically uses approximately 120–240 liters of dialysate per month—substantially less than the 240–360 liters required by standard CAPD or APD regimens. This represents an average reduction of up to 1,440 liters per patient per year.

Importantly, the environmental benefit extends beyond direct dialysate use. The water required for manufacturing the plastic components of PD systems is considerable. An Italian analysis of a CAPD-based iPD program estimated annual water savings from bag production alone at 25,056 liters, 18,144 liters, and 10,195 liters per patient for starting regimens of one, two, or three exchanges per day, respectively (30).

### Lower plastic waste generation

By reducing the number of exchanges, iPD substantially lowers the consumption of PVC dialysate bags and associated materials such as plastic tubing, connectors, cardboard packaging, and outer wrap. Early-stage iPD prescriptions have been shown to cut plastic waste by more than 50%, potentially saving hundreds of kilograms of waste per patient annually (30). In quantitative terms, switching from full-dose CAPD to an incremental approach was estimated to reduce plastic waste by 139.2 kg, 100.8 kg, and 56.6 kg per patient per year for regimens of one, two, or three daily exchanges, respectively (30). Although formal data are not yet available, the reduction in plastic production and disposal is likely even greater for patients undergoing incremental APD (15).

### Decreased carbon emissions

Life-cycle assessment models indicate that iPD can significantly reduce carbon emissions associated with dialysis. Key contributors include reduced frequency of supply deliveries, lower industrial production of consumables, and a decreased volume of waste requiring incineration—a process that is both energy-intensive and environmentally harmful.

The extent of carbon savings will depend on the specifics of the iPD regimen but given that packaging materials account for approximately 80% of PD’s carbon footprint (14), iPD could feasibly reduce dialysis-related emissions by 30–45%. Supporting this estimate, one study demonstrated that omitting a single icodextrin exchange reduced the carbon footprint of APD and CAPD by 15 and 26%, respectively (13).

## Clinical and economic advantages of incremental peritoneal dialysis

### Quality of life

Patients on incremental peritoneal dialysis may experience an improved quality of life, reduced loss of productivity, and a lower psychological burden due to fewer daily procedures. Starting PD with a reduced number of exchanges per day has been shown to increase patients’ free time. In a recent study by Nicdao et al., the total procedural time saved with one, two, or three CAPD exchanges per day was approximately 135, 90, and 45 min, respectively (46). Similarly, Nardelli et al. (30) estimated a gain of 18.1, 13.1, and 7.4 additional free days per patient-year when PD was initiated with one, two, or three daily exchanges, respectively, instead of the standard four-exchange regimen. Ultimately, the ability to initiate PD using an incremental approach may enhance treatment acceptability and, consequently, contribute to higher PD prevalence.

### Infectious risk

Incremental peritoneal dialysis theoretically carries a lower risk of peritonitis due to reduced catheter manipulation. A randomized study by Yan et al. (47) comparing incremental CAPD (three daily exchanges) and full-dose CAPD (four exchanges) in 139 incident patients showed a higher, though not statistically significant, peritonitis rate in the full-dose group (26% vs. 13%, *p* = 0.06). Similarly, studies by Sandrini and Lee (29) found no significant differences in peritonitis-free survival between incremental and standard PD. Conversely, two Asian studies reported a lower incidence of peritonitis with incremental regimens (39, 48). In an observational study, Nardelli et al. (49) reported a significantly higher risk of peritonitis in patients starting with three (HR 2.20, *p* = 0.014) or four exchanges (HR 2.98, *p* < 0.01), compared to those initiating with two. Most infections occurred within the first 12 months, highlighting this period as the most vulnerable due to inexperience with PD technique. Starting with fewer exchanges may mitigate this early risk.

### Peritoneal membrane preservation and metabolic effects

High glucose exposure in PD solutions may account for up to 35% of daily caloric intake, contributing to weight gain, hyperglycemia, dyslipidemia, and metabolic syndrome (50–57). Chronic glucose exposure also damages the peritoneal membrane, promoting angiogenesis, fibrosis, and mesothelial cell loss (58–63). Incremental PD may minimize glucose load, thus reducing systemic side effects and preserving membrane integrity (64–68).

In Nardelli et al.’s study, the estimated annual glucose exposure reduction was 20.4 kg, 14.8 kg, and 8.3 kg per patient for those starting with 1, 2, or 3 exchanges, respectively, compared to standard PD (30). While these findings suggest a potential advantage in prolonging technique survival, comparative studies have yet to demonstrate a definitive superiority of IPD over full-dose PD.

### Preservation of residual kidney function

Incremental peritoneal dialysis is believed to preserve RKF by avoiding overly aggressive dialysis during the early stages of therapy. Preserved RKF is associated with better volume control, enhanced phosphate clearance, and improved survival due to better endogenous erythropoietin and vitamin D production. Sandrini et al. (29) found significantly higher RKF at 6 months in patients starting PD with 1–2 exchanges versus standard regimens (6.2 vs. 4.5 mL/min/1.73 m^2^). A South Korean study also showed a reduced risk of anuria in the incremental group (38). Garofalo et al.’s (69) meta-analysis, which included 75,292 patients (115 on iPD), reported slower RKF decline in incremental versus full-dose dialysis (*p* = 0.007). However, these findings should be interpreted cautiously. Nardelli et al. (49) found no significant difference in RKF or urine output over 24 months between groups. Similarly, the only available RCT comparing incremental and full-dose PD showed no significant differences in GFR decline or anuria-free survival after two years (47).

### Economic considerations

Renal replacement therapy (RRT) is a major financial burden on healthcare systems. In 2022, dialysis-related Medicare expenditures in the U. S. exceeded $45.3 billion—over 6% of the total Medicare budget (70). Incremental peritoneal dialysis reduces treatment costs by requiring lower volumes of dialysis solutions and fewer exchanges. According to an Australian study, the total mean monthly outpatient cost was $1,241 per patient on incremental PD and $1,581 for fulldose PD with a mean difference of $339. The greatest contributor to the monthly cost difference was PD consumables, which was $1,190 for full dose, compared to $810 for incremental PD (46). Evaluating the cost of consumables, Nardelli et al. (30) in Italy estimated even greater annual cost savings with incremental CAPD compared to full-dose regimens. Specifically, the savings were €8,700, €6,300, and €3,540 per patient-year when initiating therapy with 1, 2, or 3 exchanges per day, respectively—reinforcing the evidence of significant financial benefits associated with lower initial prescription volumes (30).

## Barriers and enablers to incremental peritoneal dialysis

A key barrier to adopting iPD is the reluctance of patients and caregivers to escalate the dialysis dose, as doing so involves an increased number of daily exchanges and procedures. In addition, iPD necessitates close clinical surveillance of RKF to detect subtle or unpredictable declines. This requires regular timed urine collections, Kt/V urea calculations, and potentially more frequent clinic visits, increasing the workload for nephrologists and dialysis nursing teams. If RKF loss goes undetected, patients may experience inadequate solute clearance, fluid overload, and serious electrolyte imbalances. For this reason, shared decision-making is essential. Patients should be informed from the outset about the goals, advantages, and limitations of iPD. An informed patient is more likely to engage constructively in dose adjustments and adhere to dietary and fluid restrictions. Furthermore, the effectiveness of iPD in clearing middle molecules such as β2-microglobulin also warrants consideration. Unlike small solutes like creatinine, which are primarily cleared through frequent exchanges, the clearance of middle molecules is more dependent on total peritoneal dwell time (71). For example, two exchanges spread over 24 h provide nearly twice the β2-microglobulin clearance compared to the same two exchanges delivered over a 12-h period, emphasizing the possibility to tailor iPD prescriptions to solute-specific clearance goals (72). Wider implementation of iPD is also hindered by systemic challenges. These include reimbursement policies that may not accommodate incremental treatment strategies, limited provider familiarity with iPD protocols, and logistical difficulties in managing personalized and non-standard dialysis regimens. Overcoming these barriers will require targeted training programs to improve provider knowledge and confidence, policy reforms that align payment models with patient-centered care, and the adoption of digital tools and remote monitoring systems to streamline care delivery and optimize patient oversight (73–75). Despite its increased clinical complexity, iPD offers potential long-term benefits, including reduced environmental impact, cost saving, better preservation of RKF, lower risk of peritonitis, decreased glucose exposure and improved quality of life (Figure 2). These advantages may justify the added effort and resources required for its successful integration into routine clinical practice (Table 2).

## Conclusion

Incremental peritoneal dialysis is a powerful yet underutilized tool in the pursuit of sustainable kidney care. By aligning with the principles of green nephrology, iPD reduces resource consumption, medical waste, and environmental emissions, while offering safe and effective treatment to patients with residual kidney function.

As healthcare systems confront the twin challenges of climate change and chronic disease burden, adopting ecologically conscious approaches like iPD is not only advisable but necessary. Future dialysis paradigms must move beyond survival toward sustainability—and iPD offers a pragmatic, evidence-based path forward.
